# Development of Automated Reinforcement Management System (ARMS): Protocol for a Phase I Feasibility and Usability Study

**DOI:** 10.2196/25796

**Published:** 2021-07-19

**Authors:** Andre Miguel, Crystal Smith, Nicole Perea, Kim Johnson, Michael McDonell, Sterling McPherson

**Affiliations:** 1 Elson S Floyd College of Medicine Washington State University Spokane, WA United States; 2 College of Pharmacy and Pharmaceutical Sciences Washington State University Spokane, WA United States; 3 Managed Health Connections Spokane, WA United States

**Keywords:** alcohol use disorder, contingency management, ecological momentary assessment, treatment

## Abstract

**Background:**

Alcohol use is directly related to over 3 million deaths worldwide every year. Contingency management is a cost-effective treatment for substance use disorders; however, few studies have examined its efficacy for alcohol use disorder. Recent technological advances have enabled the combined use of mobile apps and low-cost electronic breathalyzer devices to remotely monitor alcohol use. Leveraging this type of technology, our study group has recently developed an integrated contingency management system that would enable community treatment programs to remotely deliver contingency management to anyone who owns a smartphone.

**Objective:**

In this paper, we present a full description of our integrated contingency management system, Automated Reinforcement Management System (ARMS), and describe a protocol that will evaluate its feasibility and usability.

**Methods:**

Initially, 6 clinicians will participate in a 1-hour focus group where the study staff will navigate through ARMS as it would be used by clinicians and patients. Clinicians will provide feedback on the intervention in general, which will be used to modify ARMS to make it more user friendly, time saving, and relevant to treatment. A second focus group will summarize the changes made following the initial clinician feedback and will provide additional input regarding the potential utilization of ARMS. Thereafter, the clinicians’ acceptability of ARMS will be evaluated using the System Usability Scale. Following the clinicians’ assessments of ARMS and final modifications, the system will be evaluated in terms of feasibility and patient usability by using an A-B-A within-subject experimental design wherein 20 treatment-seeking individuals with alcohol use disorder will be recruited. The two A phases (control conditions) will each last 2 weeks, and the B phase (contingency management condition) will last 4 weeks. During all phases, participants will be asked to use the ARMS app to submit three breathalyzer samples per day (at 10 AM, 2 PM, and 8 PM). Participants will be prompted by the ARMS app at these predetermined times to record and submit their breathalyzer samples. During the A phases, participants will earn vouchers for every breathalyzer sample submitted, independent of their sample results. During the B phase, vouchers will be provided contingent upon the submission of alcohol-negative breathalyzer samples (breath alcohol content = 0.00). At the end of the A-B-A experiment trial, patients’ usability of the ARMS app will be evaluated using the System Usability Scale. Feasibility will be measured based on whether the ARMS app helped significantly increase alcohol abstinence.

**Results:**

Recruitment for this study began in January 2021 and is expected to be completed by December 2021.

**Conclusions:**

This study will provide the baseline capability for the implementation of a remotely monitored contingency management platform. If successful, ARMS has the potential to provide effective treatment for alcohol use disorders to individuals living in remote rural areas.

## Introduction

Alcohol use is directly related to over 3 million deaths worldwide each year (ie, 5.3% of all deaths reported annually) and is a casual factor of more than 200 diseases, injuries, and conditions [1]. In the United States, the lifetime and past-year prevalence of alcohol use disorder (AUD) are 29.1% and 13.9%, respectively [2], making it the most prevalent substance use disorder in the country [3].

Contingency management is an effective treatment for substance use disorders that consists of providing reinforcement (ie, rewards) after an objective verification of drug abstinence through the submission of a substance-negative biospecimen [4-6]. Although the efficacy of contingency management has been demonstrated for a range of substance use disorders [7-9], fewer studies have examined the efficacy of contingency management for AUD [10-14]. This was primarily due to the lack of biomarkers that could detect alcohol consumption for prolonged periods. Until recent years, the only commercially available biomarker for alcohol use was breath alcohol content (BAC), assessed using breathalyzers that measure the concentration of ethanol in an individual’s breath [15,16]. Although accurate, this biomarker can only detect alcohol use for up to 12 hours after consumption; moreover, when used infrequently, it is a tool better used to assess intoxication rather than abstinence [14]. As a result, to accurately assess recent alcohol consumption, contingency management interventions using a breathalyzer require individuals to be physically present at an office or treatment center to submit samples multiple times per day [14], limiting the feasibility of such an approach.

Fortunately, recent technological advances have enabled the combined use of mobile apps and low-cost consumer electronic breathalyzer devices to remotely monitor alcohol use [17,18]. Studies wherein smartphone apps and remote breathalyzers were used to provide contingency management for AUD have found them to be effective in promoting alcohol abstinence [17,19,20]. Although other technologies, such as prescription digital therapeutics (eg, reSET and reSET-O by Pear Therapeutics), have been approved by the US Food and Drug Administration (FDA) for substance use disorders, they have not directly targeted substance abstinence. As a result, to date, app-based contingency management technologies for alcohol use have yet to be integrated into AUD treatment programs and evaluated in community treatment settings.

To further advance research on the use of technology to address AUD and to enable the development of more effective treatment strategies, our research group has partnered with Managed Health Connections [21] to develop an integrated, end-to-end contingency management system that would enable community treatment programs to deliver contingency management remotely to anyone who owns a smartphone and seeks to reduce their alcohol consumption. In this methods paper, we present a full description of an integrated contingency management system, termed Automated Reinforcement Management System (ARMS), and describe the protocol that will evaluate its feasibility and usability.

## Methods

### Participants

This study will be conducted within the community and with community clinic partners in Spokane, WA, USA, under the auspices of the Washington State University Program of Excellence in Addictions Research at the Analytics and PsychoPharmacology Laboratory. In this phase 1 study, a total of 6 clinicians and 20 patients will be recruited. Patients of either biological sex who are 18 years of age or older and have an Alcohol Use Disorders Identification Test (AUDIT) [22] score of 8 or higher will be eligible to enroll in the study. Participants must read and speak English and have the ability to provide written informed consent. Patients with severe AUD or a co-occurring psychotic disorder according to the Diagnostic and Statistical Manual of Mental Disorders, Fifth Edition (DSM-V) [23], with significant risk of dangerous alcohol withdrawal (defined as a history of alcohol detoxification or seizure in the last 12 months and expression of concern by the individual about dangerous withdrawal), or with a lifetime suicide attempt or suicidality in the past year will not be eligible for enrollment in the study.

### Study Design

To evaluate the feasibility and usability of ARMS delivered in treatment settings, our research team has designed a three-step study where ARMS will be evaluated by both treatment providers and patients. Initially, 6 clinicians will participate in a 1-hour-long interview or focus group (depending on their availability) that will describe and provide visuals of the contingency management intervention and introduce patient- and provider-facing apps. Study staff will navigate through the program in ways consistent with both clinician and patient users. Clinicians will also participate in a semistructured interview where they will be asked to provide information on their standard protocol when working with patients who have AUD, such as how they currently track progress and communicate with patients. Furthermore, they will be asked to provide feedback on the intervention in general, including why they would or would not use the app, suggestions for improvement, app usability, potential obstacles that may be encountered with the use of the app, preferences for accessing the data generated by the app, and preferences on which data they would prefer to have highlighted briefly.

The information provided by the clinicians in this first focus group will then be used to modify ARMS to make it more user friendly, time saving, and relevant to treatment (possibly by incorporating new features that may assist treatment). A second focus group will then summarize the changes made following the clinician feedback and provide deidentified user data allowing clinicians to navigate through the system and provide additional feedback regarding the potential utilization of patient data.

Once the modifications are made in response to the second wave of clinician feedback, ARMS will be evaluated primarily for efficacy but also for feasibility and patient usability through an A-B-A (or, “return-to-baseline”) completely within-subject experimental design. Following similar single subject-design studies conducted by our group [20,24], the two A phases will each last 2 weeks and the B phase will last 4 weeks, for a total study duration of 8 weeks. During all phases, participants will be asked to use the ARMS app to submit breathalyzer samples at 10 AM, 2 PM, and 8 PM each day. Submission of each sample should take less than 5 minutes. Participants will be prompted by their ARMS app to record and submit their breathalyzer samples at these predetermined times and will have a half-hour window following the prompt to submit each sample. During the A phases (control conditions), participants will earn US $2 in vouchers for every breathalyzer sample submitted during the predetermined period, independent of their sample results. Notably, this reward system will be in effect for both the first and second A phases. During the B phase (contingency management condition), vouchers will be provided contingent upon the submission of alcohol-negative breathalyzer samples—that is, breath alcohol content (BAC) = 0.00. Participants will begin by receiving vouchers worth US $2 per alcohol-negative breath sample submitted, with the value escalating by US $0.25 per alcohol-negative sample submission to a maximum value of US $3.50 per submission. Vouchers will be reset to US $2 per sample if a participant does not submit a sample in the predetermined period, if an alcohol-positive breath sample is submitted, or if the participant cannot be identified from the facial recognition photo (see details below).

At the end of the A-B-A experiment trial, the patients’ acceptability of ARMS will be evaluated with the System Usability Scale (SUS).

### Assessments

Assessments collected in this study will include sociodemographic and medical or treatment history data, the Mini-International Neuropsychiatric Interview [25], Addiction Severity Index-Lite (ASI) [26], Brief Symptom Inventory (BSI) [27], DSM-V [23], Fagerström Test for Nicotine Dependence [28], AUDIT [22], Alcohol Urge Questionnaire (AUQ) [29], and 15D Health-Related Quality of Life (HRQoL) [30]. Alcohol use will be measured using the Alcohol Timeline Follow Back (ATFB) method [31], and BAC results will be collected via the ARMS app. Both the providers’ and patients’ acceptability toward ARMS will be evaluated using the SUS [32]. For the assessment schedule, see Table 1.

**Table 1:** Assessment schedule for the A-B-A design experimental trial.

Content	Baseline	A-B-A							
	Week 0	Week 1	Week 2	Week 3	Week 4	Week 5	Week 6	Week 7	Week 8
Informed consent	✓								
Sociodemographic	✓								
Medical/treatment history	✓								
DSM-V^a^	✓								
Mini-International Neuropsychiatric Interview	✓								
Addiction Severity Index: ASI-Lite	✓		✓		✓		✓		✓
15D Health-Related Quality of Life	✓		✓		✓		✓		✓
Alcohol Urge Questionnaire	✓	✓	✓	✓	✓	✓	✓	✓	✓
Fagerström Test for Nicotine Dependence	✓		✓		✓		✓		✓
Alcohol Use Disorders Identification Test (AUDIT)	✓	✓	✓	✓	✓	✓	✓	✓	✓
Brief Symptom Inventory (BSI)	✓		✓		✓		✓		✓
Alcohol use (biochemical BAC^b^)	✓	3 times per day							
Alcohol Timeline Follow Back (self-report)	✓	✓	✓	✓	✓	✓	✓	✓	✓
Patient acceptability: System Usability Scale		✓	✓	✓	✓	✓	✓	✓	✓

^a^DSM-V: Diagnostic and Statistical Manual of Mental Disorders, Fifth Edition.

^b^BAC: blood alcohol content.

### ARMS Mobile App Intervention

ARMS is a hybrid mobile/IOS web-based app for contingency management designed to integrate with the electronic medical record of treatment programs, providing clinicians with an adjunctive treatment modality to (1) administer contingency management treatment to patients with AUD remotely, (2) access updated patient information that can be critical to the clinical management of AUD, and (3) communicate safely and directly with patients through the ARMS app. ARMS includes two separate interfaces: (1) a mobile/IOS interface to be used by patients and (2) a web-based application to be used by treatment providers.

### Patient Interface and Dashboard

The patient interface includes five features: (1) recording and submitting breathalyzer samples, (2) recording and submitting ecological momentary assessments (EMAs) on moods and executive function that may be pertinent to treatment, (3) reminders and messages, (4) redemption of rewards, and (5) a patient dashboard (see below for more details). To comply with the Health Insurance Portability and Accountability Act requirements, ARMS uses a user-protected login for access.

### Breathalyzer Sample Recording and Submission

A critical aspect of the contingency management component involves providing rewards contingent upon the objective verification of alcohol abstinence. In ARMS, the patient mobile interface syncs via Bluetooth with a BACtrack breathalyzer device, enabling the recording and submission of breathalyzer sample results from the BACtrack device to the ARMS app. The app prompts users, instructing them to place their face in the center of a square displayed on their phone screen while blowing into the breathalyzer. A photo is then captured to allow for facial recognition of the patient submitting the sample (ie, a full-face image is required to conduct the facial recognition). Immediately after the breathalyzer result is captured, the ARMS app presents both the breathalyzer result and the facial image and provides the patient the option to submit the breathalyzer result to their provider. If the patient agrees with the breathalyzer result and believes that the captured image will allow them to be identified, they can click on “submit” and the breathalyzer result will be uploaded onto a secure server accessible only by their treatment providers and the study researchers. If the patient does not agree with the breathalyzer result or believes that the photo will not allow for facial recognition, the option to submit another sample can be chosen.

### Ecological Momentary Assessment

After the submission of the breathalyzer result, ARMS will prompt the patient to respond to 3 to 5 questions about moods and states that might be pertinent to the patient’s recovery. As shown in Table 2, the ecological momentary assessment (EMA) questions include options that were created based on the Addiction Neuroclinical Assessment (ANA) framework developed by Kwako et al [33,34], which postulates that three domains defined as poor executive functioning (ie, working memory and impulsivity), negative emotionality (eg, depression, anxiety, and symptoms of withdrawal), and incentive salience (eg, thinking about and/or craving alcohol) are the primary factors that cause and maintain addictions [33-37]. Kwako and colleagues [33] propose that ANA domains, assessed by self-reports and cognitive testing, as well as genetic analysis and, eventually, neuroimaging, can be used to identify groups of patients who do not respond to existing treatments. The ANA also provides a framework to guide treatment adaptations to improve outcomes for specific groups (ie, nonresponders or more severe disease groups). Furthermore, it can be used to match individuals to specific interventions based on their ANA characteristics. Thus, the ANA may provide an ideal structure for developing a personalized medicine approach to integrated treatment for AUD.

**Table 2:** Description of the ecological momentary assessment (EMA) questions.

EMA question	Type of response	ANA^a^ domain
Since your last assessment, how much have you craved alcohol?	5-point Likert scale	Reward salience
I want to drink so bad I can almost taste it.	5-point Likert scale	Reward salience
How happy do you feel right now?	5-point Likert scale	Negative emotionality
How sad do you feel right now?	5-point Likert scale	Negative emotionality
How relaxed do you feel right now?	5-point Likert scale	Negative emotionality
How Stressed do you feel right now?	5-point Likert scale	Negative emotionality
How bored do you feel right now?	5-point Likert scale	Negative emotionality
How irritable do you feel right now?	5-point Likert scale	Negative emotionality
In the last two hours have you been doing things without thinking?	5-point Likert scale	Executive functioning
In the last two hours have you been acting on impulse?	5-point Likert scale	Executive functioning
In the last two hours have you felt self-controlled?	5-point Likert scale	Executive functioning

^a^ANA: Addiction Neuroclinical Assessment.

The EMA questions in ARMS will provide clinicians and researchers with relevant real-time information about their patients’ states and moods specific to the three ANA domains. This information may serve multiple purposes. First, comparing the EMA answers and breathalyzer results of the general patient population may assist clinicians in identifying specific traits and states associated with positive or negative treatment outcomes, possibly enabling them to identify, at the beginning of treatment, whether a participant may benefit from more intensive care. Second, and perhaps more important, by comparing the EMA answers and breathalyzer results of a specific patient, clinicians may be able to identify specific cognitions and moods associated with risks of future relapse, enabling them to intervene before relapse occurs.

### Reminders and Messages

**Figure 1:**
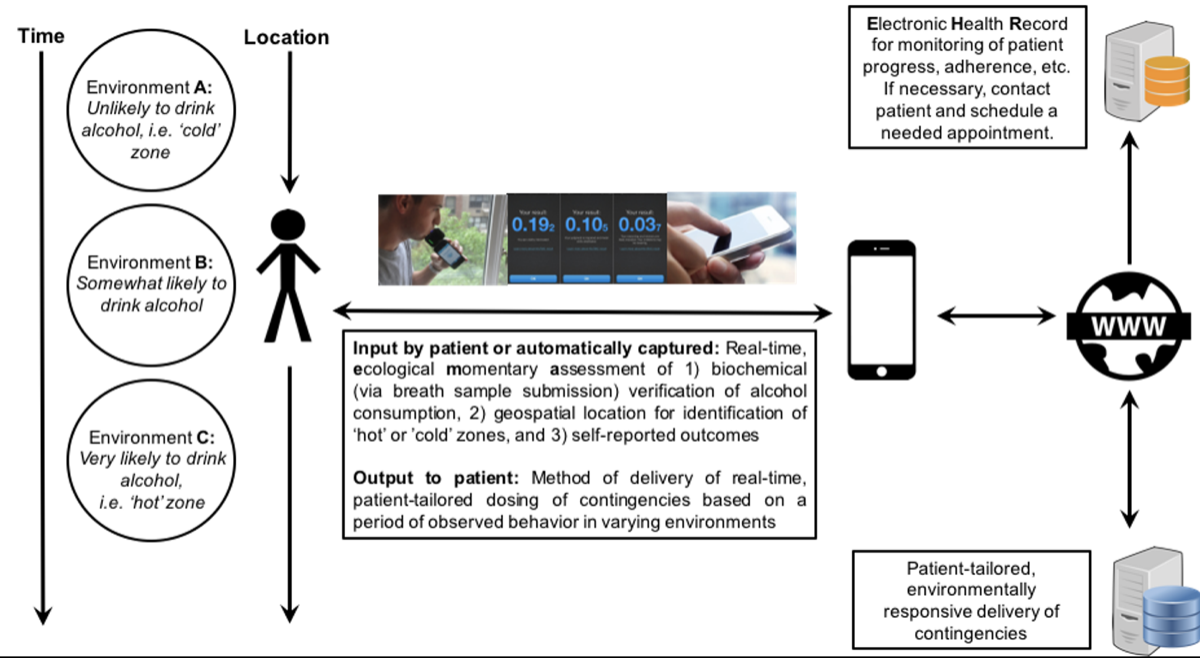
Data capture and participant flow.

To promote compliance and recovery, the ARMS app will send reminders when it is time to submit the breathalyzer samples and EMA responses. It will also send an alert when patients fail to submit a sample. To further enhance treatment response, ARMS will also send reinforcing messages. These messages range from congratulating patients when they reach a certain milestone (eg, “Three straight days of alcohol abstinence, keep up the good work!”) to encouraging them after relapse (eg, “Treatment success is not about falling but how quickly we pick ourselves up” and “Tomorrow you get another chance to do what’s right by you!”). Patients will also receive messages confirming their breathalyzer sample submissions and facial image validations, as well as messages informing them about the number of rewards earned in the contingency management intervention. Figure 1 presents an overall schematic of how ARMS functions.

### Redeeming Rewards

Patients will be able to use the ARMS app to redeem the credit earned immediately after the breathalyzer sample is validated. Patients can choose to redeem any specific amount in the form of a digital gift card sent to a preregistered email address. The gift card information will also be stored in both the patient’s and provider’s ARMS interfaces.

### Provider Interface

All data collected in the patient’s mobile ARMS app will be stored securely in a database that can be accessed by providers from the provider dashboard. This web-based console allows providers to register new patients, access and evaluate patient performance, and validate the authenticity of the breathalyzer samples submitted. Furthermore, the provider dashboard provides an easy way for providers to identify patients that may need more personalized care by displaying a summary of the status of all patients enrolled in the system, with nonadherent patients or patients failing to achieve their goals being placed at the top of the list. The ARMS provider interface was also created to integrate directly into electronic medical record systems using standard health information exchange formats, such as Health Level Seven (HL7; a specific method for the exchange, integration, sharing, and retrieval of electronic health information) and Clinical Core Document (which offers updates to previous efforts by HL7), thereby enabling clinicians to receive progress alerts based on nonadherence and overall status. These provider alerts are customizable to accommodate variations in workflow.

### ARMS Contingency Management Intervention

Contingency management interventions involve providing reinforcement (ie, rewards or incentives) after a targeted behavior is exhibited. In the field of substance use disorder treatment, contingency management usually consists of providing vouchers of monetary value after a specific period of substance abstinence is verified through the submission of a negative drug screening sample. The combination of features present in ARMS and the BACtrack breathalyzer device enables the provision of contingency management remotely to any patient that owns a smartphone. To provide more personalized care, the ARMS contingency management intervention is flexible, allowing providers to modify and adjust several components of the intervention. This flexibility allows providers to mold the contingency management intervention to best suit the patient’s specific needs as well as to adapt it to the daily clinic routine.

To initiate the contingency management intervention with ARMS, treatment providers will select the type of response that will be targeted in that intervention (eg, alcohol abstinence and low levels of alcohol use), the schedule of reinforcement (ie, the number of samples to be submitted daily), and the duration of the intervention. After these parameters are set, patients will be prompted by their ARMS app to record and submit their breathalyzer samples at the predetermined times. To reduce the burden or other inconveniences related to sample submission, patients will have a half-hour window to submit each sample following the prompt. After a sample is submitted, patients will receive a message stating that the sample was received and is currently awaiting verification. A research staff member will then have 24 hours to confirm the patient’s identity (based on the facial image captured at the time of sample collection). The research staff member will be presented with a recommendation for approval or rejection based on facial recognition verification. This process will be evaluated for the potential to automate verification. After providers verify the authenticity of the sample, patients will be immediately informed that the sample was verified and, when the target response is achieved (ie, confirmed alcohol abstinence), patients will be notified of the number of rewards earned. If the sample is deemed invalid or the target behavioral response is not accomplished, patients will be notified of the reason they did not earn the reward.

### Buildout Options

There are additional features that are either in progress or are planned for future iterations. One such feature is to enable the selection of different target responses such as abstinence (ie, BAC=0.00) or reduction of heavy drinking (ie, BAC=0.08). We also plan to enable the selection of different options related to specific components of the contingency management intervention; for example, setting the expected number of sample submissions to be fewer than 3 submissions per day, scheduling sample requests at different times of day, or using artificial intelligence to conduct immediate facial recognition after the BAC sample is submitted**.** We also intend to make reminders multimodal so that they can reach users through different means based on their preferences. Users would thus be able set up their reminders as local alarms on their smartphone device or receive them via push, SMS text messages, or automated voice calls.

Another feature that is in progress but will not be active during this phase 1 study is defining patient-specific “hot zones” (and “cold zones”) using a type of geolocation “fence” in an effort to provide patients with feedback and alert them when they are entering a physical area or location where they have not achieved their stated drinking goals (Figure 1). Finally, we plan to develop additional dashboard features, including the ability for patients to see which EMA responses are correlated with alcohol outcomes over time and which factors are most likely associated with craving and alcohol use, to promote additional awareness of executive function and moods associated with relapse.

### Outcomes

Patient usability will be evaluated using the SUS [32] collected at the end of the A-B-A experimental trial. The use of SUS has been recently recommended by the FDA, and it has been used to evaluate the usability of similar technologies. For this study, a nominal SUS score of 68 or higher will be considered as our target score. The feasibility of ARMS will be measured by whether ARMS could significantly increase alcohol abstinence. Other outcomes will include patient adherence to and compliance with the ARMS app considering (1) the proportion of participants that complete the experiment, (2) the proportion of breathalyzer samples submitted, and (3) the proportion of EMA questions answered during the trial. Additional secondary outcomes will include the efficacy of ARMS considering (1) self-reported alcohol use (ATFB), (2) levels of alcohol craving (AUQ), and (3) overall life-functioning (ASI, BSI, and HRQoL). Finally, we will explore whether and how the ANA framework responses collected thrice daily with EMA correlate with alcohol use or abstinence. For instance, we expect that the information captured with the EMA and breathalyzer results will allow the identification of specific mood and state traits that may predict future relapse and, thus, enable the development of personalized relapse prevention strategies.

## Results

Recruitment for this study began in January 2021. Data collection will be finalized by December 2021, and we expect to publish our results by the second semester of 2022.

## Discussion

### Principal Findings

There currently exists a gap in our knowledge of how to best treat people who wish to reduce their alcohol use, especially in a real-time manner and among those dwelling in areas where access to in-person treatment may be challenging. It is well understood that contingency management is one of the most effective interventions for substance use disorders; however, contingency management has not been widely applied in the field of AUD treatment, in part, because of the difficulty of detecting alcohol abstinence using standard breath alcohol test procedures [15,38]. Our study capitalizes on recent technological advances and clear collaboration potential between academia and start-up companies to partner and develop a smart phone-based contingency management application enabling remote delivery adjunctive treatment for individuals seeking outpatient treatment for AUD.

This phase 1 project will provide baseline capability for the implementation of a remotely monitored contingency management platform. The clinician and user feedback will provide insight into enhancements that can increase utilization and effectiveness. Future capabilities may include (1) adaptation of report-back thresholds to address heavy drinking in addition to abstinence, (2) customization of report-back windows to accommodate user schedules, (3) multimodal reminders (SMS, email, etc) for users and trusted caregivers, (4) geolocation-related alcohol use reporting, and (5) enhanced feedback based on correlations of multiple data points with alcohol use. After completion of this study, we plan to have the necessary information to develop a phase-2 effectiveness trial.

### Limitations

This study has some potential limitations that should be noted. First, in this current stage, ARMS was developed to be used in iOS only. Although this decision was decided strictly due to our budget, we intend to build ARMS for Android as part of our phase 2 trial. Second, this study did not include formal focus groups with the participating patients to evaluate the acceptability and suggestions for improvements by participants. We intend to incorporate such focus groups as part of our phase 2 trial.

## Abbreviations

ANA: addiction neuroclinical assessment
ARMS: Automated Reinforcement Management System
ASI: Addiction Severity Index-Lite
ATFB: Alcohol Timeline Follow Back
AUD: alcohol use disorder
AUDIT: Alcohol Use Disorders Identification Test
AUQ: Alcohol Urge Questionnaire
BAC: blood alcohol content
BSI: Brief Symptom Inventory
DSM-V: Diagnostic and Statistical Manual of Mental Disorders, Fifth Edition
EMA: ecological momentary assessment
FDA: Food and Drug Administration
HL7: Health Level Seven
HRQoL: 15D Health-Related Quality of Life
SUS: System Usability Scale
